# The Wnt11 Signaling Pathway in Potential Cellular EMT and Osteochondral Differentiation Progression in Nephrolithiasis Formation

**DOI:** 10.3390/ijms160716313

**Published:** 2015-07-17

**Authors:** Deng He, Yuchao Lu, Henglong Hu, Jiaqiao Zhang, Baolong Qin, Yufeng Wang, Shuai Xing, Qilin Xi, Shaogang Wang

**Affiliations:** 1 Department of Urology, Tongji Hospital of Tongji Medical College of Huazhong University of Science and Technology, Wuhan 430030, Hubei, China; E-Mails: hedeng2002@163.com (D.H.); luyuchao@hust.edu.cn (Y.L.); m201275806@hust.edu.cn (H.H.); medzjq@163.com (J.Z.); qinbaolong@hust.edu.cn (B.Q.); yfwangtjm@163.com (Y.W.); 2 Department of Gastroenterology, Tongji Hospital of Tongji Medical College of Huazhong University of Science and Technology, Wuhan 430030, Hubei, China; E-Mail: xingshuai529378226@126.com; 3 Department of Urology, the First Affiliated Hospital of Soochow University, Suzhou 215006, Jiangsu, China; E-Mail: linqix_827@163.com

**Keywords:** TGF-β1, calcium channel, Wnt11, nephrolithiasis

## Abstract

The molecular events leading to nephrolithiasis are extremely complex. Previous studies demonstrated that calcium and transforming growth factor-β1 (TGF-β1) may participate in the pathogenesis of stone formation, but the explicit mechanism has not been defined. Using a self-created genetic hypercalciuric stone-forming (GHS) rat model, we observed that the increased level of serous/uric TGF-β1 and elevated intracellular calcium in primary renal tubular epithelial cells (PRECs) was associated with nephrolithiasis progression *in vivo*. In the setting of high calcium plus high TGF-β1 *in vitro*, PRECs showed great potential epithelial to mesenchymal transition (EMT) progression and osteochondral differentiation properties, representing the multifarious increased mesenchymal and osteochondral phenotypes (Zeb1, Snail1, Col2A1, OPN, Sox9, Runx2) and decreased epithelial phenotypes (E-cadherin, CK19) bythe detection of mRNAs and corresponding proteins. Moreover, TGF-β-dependent Wnt11 knockdown and L-type Ca^2+^ channel blocker could greatly reverse EMT progression and osteochondral differentiation in PRECs. TGF-β1 alone could effectively promote EMT, but it had no effect on osteochondral differentiation in NRK cells (Rat kidney epithelial cell line). Stimulation with Ca^2+^ alone did not accelerate differentiation of NRK. Co-incubation of extracellular Ca^2+^ and TGF-β1 synergistically promotes EMT and osteochondral differentiation in NRK control cells. Our data supplied a novel view that the pathogenesis of calcium stone development may be associated with synergic effects of TGF-β1 and Ca^2+^, which promote EMT and osteochondral differentiation via Wnt11 and the L-type calcium channel.

## 1. Introduction

Nephrolithiasis remains one of the most common and highly recurrent urological diseases worldwide [1]. The complicated mechanism of nephrolithiasis has not been fully elucidated to date yet. Traditionally, the biochemical basis for stone formation is supersaturation of urine with relation to the stone minerals, involving increased supersaturation of calcium and oxalate [2]. Recent data reported that as many as 30%–50% of male stone formers investigated in stone clinics have hypercalciuria, which in most cases is idiopathic [3]. To our knowledge, idiopathic hypercalciuria (IH) is the finality of the interaction between genetic background and environment. IH may involve the dysregulation of multiple calcium transport systems, including increased intestinal absorption of calcium, primary renal leak of calcium (decreased renal calcium reabsorption) and increased bone demineralization [4,5].

Calcium, as a highly versatile intracellular messenger, is responsible for regulating numerous cellular functions [6]. The variations between intracellular Ca^2+^ and extracellular Ca^2+^ have great significance on making mesenchymal stem cells (MSCs) differentiate into several cell types [7,8]. Physiologically, MSCs are initiated by autocrine/paracrine ATP through the stimulation of the inositol trisphosphate receptors (IP3Rs), which mediated Ca^2+^ and are sustained by Ca^2+^ influx/extrusion through the cell membrane [9]. Moreover, Ca^2+^ can initiate gene expression in hBMSCs and regulate its proliferation, differentiation and mineralization via Ca^2+^ oscillations [10]. We then speculated that Ca^2+^ participating in the pathogenesis of renal stones may depend on the changes of epithelial cell phenotype.

Transforming growth factor βs (TGF-βs) plays significant roles in various biological effects, including cell differentiation, proliferation, apoptosis and regulating the growth, development and regeneration of extracellular materials. The function of the profibrotic cytokine TGF-β1 in the initiation and progression of renal fibrosis has been intensively studied. Previous studies showed that TGF-β1 may play an important role in epithelial to mesenchymal transition (EMT) according to the phenomena of renal tubular epithelial cells’ epithelial phenotype loss (e.g., E-cadherin) and the new mesenchymal characteristic phenotype (e.g., Zeb1, Snail1) induced by TGF-β1 [11]. Transforming growth factor-β (TGF-β) binds to and activates the cell surface receptor complex, which is comprised of receptor types I (TβRI) and II (TβRII), to phosphorylate receptor-bound Smad (Smad2/3) transcription factors [12]. Phosphorylated Smad2/3 forms a heteromeric complex with Smad4 and then translocates to the nucleus to activate or repress target gene expression, but their targets and functions are distinct under various cellular contexts. Additionally, the Wnt signaling pathways have also been linked to TGF-β1 and to EMT during such diseases; components of the Wnt signaling pathways are activated by TGF-β1. Among these process, the non-canonical signaling protein Wnt11 is directly regulated by TGF-β1 through Smad3 in both primary and immortalized renal epithelial cells. Conversely, Wnt11 also enhances the effects of TGF-β1 and is necessary for maximal activation of mesenchymal genes, such as Zeb1 and Snail1 [13]. Moreover, Wnt signaling is an upstream regulator of bone morphogenetic protein (BMP) signaling; activation of Wnt signaling induces differentiation of pluripotent mesenchymal cells into osteoblast progenitors that become osteoblasts and maintains the precursor status of these osteoprogenitors. BMP signaling stimulates these cells to further differentiate into mature osteoblasts Wnt/β-catenin signaling pathways upstream, cooperatively regulating osteoblast differentiation and bone formation. Nevertheless, little is known about the function of the non-canonical Wnt11 signaling pathway in mechanisms of stone formation and its relation to TGF-β1.

Our previous study suggested that bone formation-related proteins, such as BMP2 and osteopontin (OPN), may play an important role in renal stone formation in the IH model [14]. In addition, BMP2, OPN and the vitamin D (1,25-dihydroxyvitamin D3) receptor (VDR) are also upregulated in renal specimens of IH patients; co-incubation of TGF-β1 and calcium significantly increased the expression levels of these factors in primary renal tubular epithelial cells (PRECs) of IH patients [15]. Thus, we hypothesized that a high intracellular calcium level greatly enhances TGF-β1-induced EMT via the activation of L-type Ca^2+^ channels and the Wnt11 signaling pathway in PRECs from genetic hypercalciuric stone-forming rats as an *in vitro* model of IH. In the present study, we aim to investigate whether blockage of L-type Ca^2+^ channels or Wnt11 could significantly attenuate the EMT process in an IH rat model and further study the effects of calcium and TGF-β1 on cellular differentiation in NRK cells.

## 2. Results

### 2.1. Basal Levels of TGF-β1 in GHS Rats and Intracellular Ca^2+^ Concentration in Isolated PRECs

**Figure 1:**
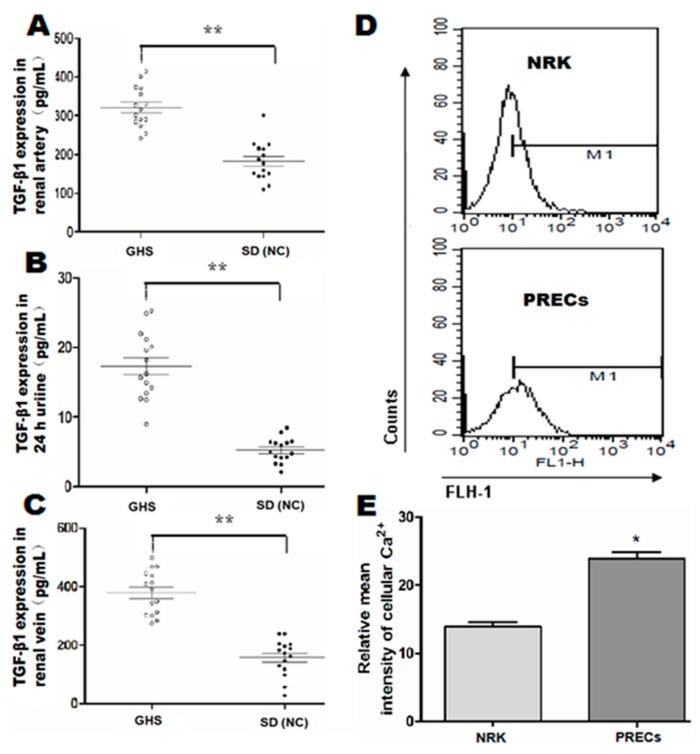
Quantitative analysis for baseline levels of cytokines TGF-β1 in renal serum and 24-h urine samples in genetic hypercalciuric stone-forming (GHS) rats and calcium ion concentrations in isolated primary renal tubular epithelial cells (PRECs). TGF-β1 in renal artery (**A**), 24-h urine (**B**) and renal vein (**C**) was analyzed by enzyme-linked immunosorbent assay (ELISA) using rat ELISA kits in fifteen GHS rats comparing with corresponding TGF-β1 contents in fifteen age- and weight-matched normal control (NC) Sprague Dawley rats, respectively. ** *p* < 0.01; (**D**) representation of intracellular Ca^2+^ concentration detection by flow cytometric analysis in isolated PRECs from GHS rats and corresponding normal control NRK cells; (**E**) mean intensity of Fluo-3/AM fluorescence in the PRECs and NRK groups (*n* = 5). * *p* < 0.05.

Previous research [16] mentioned that genetic hypercalciuric stone-forming (GHS) rats were the best animal model for studying idiopathic hypercalciuric (IH) nephrolithiasis pathogenesis, because of their many similar characteristics with IH patients. A colony of GHS models established by our panel showed consistently that hypercalciuria approximately excretes 5–8-times as much calcium as control rats. We compared the expression levels of TGF-β1 in renal blood and 24-h urine between GHS rats and SD normal controls rats (NC). Significantly, we found that TGF-β1 expression in renal artery and vein were increased in GHS rats (*n* = 15, Figure 1A,C) (*p* < 0.01). A similar trend was also observed in terms of 24-h urine TGF-β1 in GHS rats (*n* = 15, Figure 1B) (*p* < 0.01). In addition, we observed that the intracellular Ca^2+^ concentration in GHS rats was also significantly increased (*p* < 0.05, Figure 1D,E).

### 2.2. Different Expression of Specific Bio-Markers in EMT and Osteochondral Differentiation between PRECs and NRK Cells

**Figure 2:**
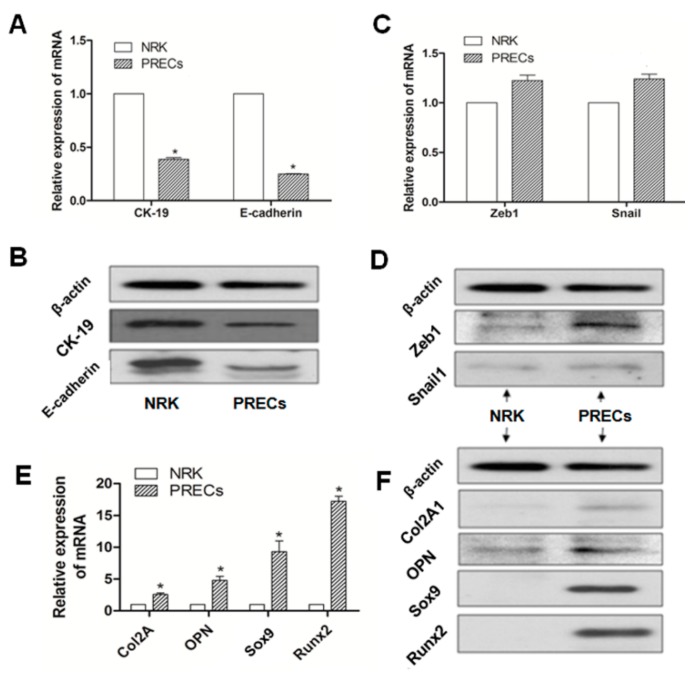
Representation of the relative mRNA (**A**) and protein expression (**B**) of epithelial phenotypic markers (E-cadherin, CK19); MSC phenotypic markers (Zeb1, Snail1) (**C**,**D**); and osteochondral markers (Col2A1, OPN, Sox9, Runx2) (**E**,**F**); in PRECs compared with NRK cells, * *p* < 0.05 (*n* = 3).

To illustrate whether the EMT process and potential osteochondral differentiation were involved in PRECs in the model of GHS rats, PRECs and NRK cells were obtained, and then, specific bio-markers, including epithelial phenotypic markers (E-cadherin, CK19), MSC phenotypic markers (Zeb1, Snail1) and osteochondral markers (Col2A1, OPN, Sox9, Runx2), were examined by real-time RT-PCR and Western blot, respectively. Expressions of E-cadherin and CK19, in terms of mRNA or protein, were significantly downregulated in PRECs compared with those in NRK cells (*p* < 0.05) (Figure 2A,B). Although there was no statistical significance, gene and protein (Figure 2C,D) expression levels of Zeb1 and Snail1 were slightly increased in PRECs compared with those in the control. Those data indicated that a transient mesenchymal state may exist in stone formation related to EMT in GHS rats. Further, we examined the osteogenic (Col2A1, Sox9) and chondrogenetic (OPN, Runx2) markers; The results revealed that all of these markers, according to mRNA or protein level, were significantly increased in PRECs (Figure 2E,F), compared with those levels in NRK (*p* < 0.05).

### 2.3. Wnt11 Knockdown Attenuated the Expression of Osteogenic/Chondrogenetic Factors and Reversed the EMT Process in PRECs

**Figure 3:**
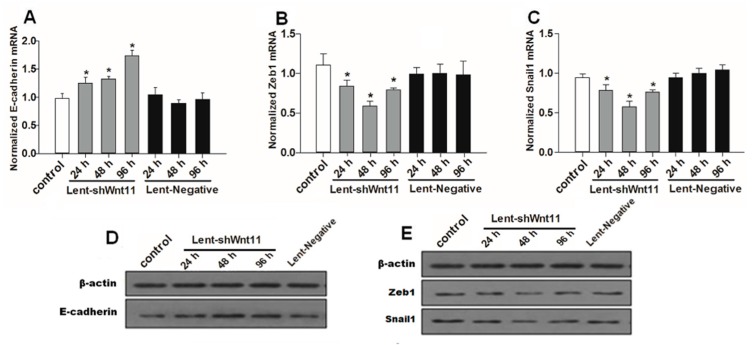
Wnt11 knockdown reversed the potential of the EMT process in PRECs. Quantitative detection for mRNA levels of E-cadherin (**A**); mesenchymal marker Zeb1 (**B**); Snail1 (**C**) in PRECs with Wnt11 knockdown. The data represent the mean ± SD of at least three independent experiments; * *p* < 0.05 compared with the control. Western blot (**D**,**E**) showed a similar trend in protein expression levels as in mRNA levels after silencing the Wnt11 gene in PRECs.

**Figure 4:**
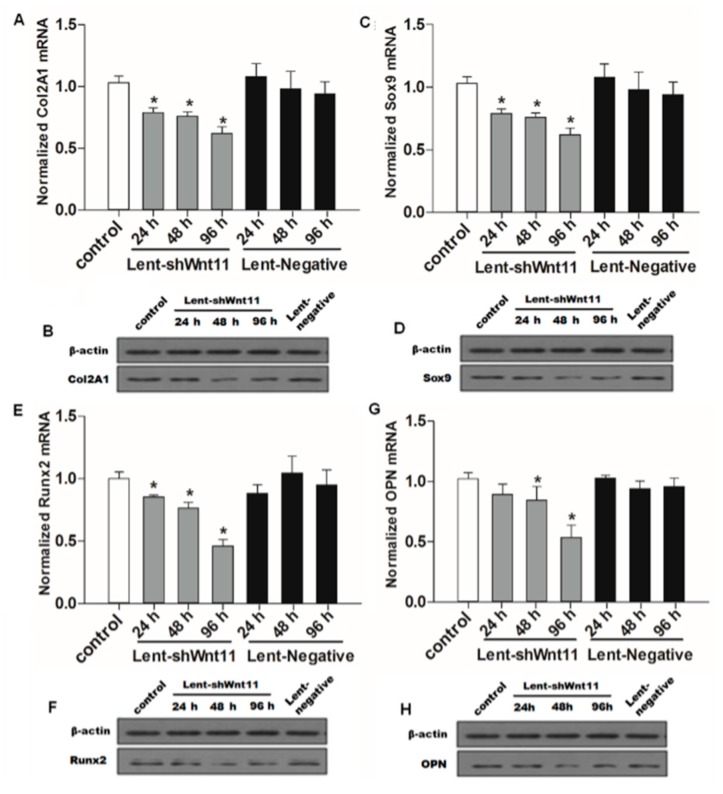
Wnt11 depletion reversed the potential of the osteochondral differentiation in PRECs. Quantitative and qualitative analysis for mRNA and protein levels of osteochondral markers Col2A1 (**A**,**B**); Sox9 (**C**,**D**); Runx2 (**E**,**F**) and OPN (**G**,**H**) in PRECs with Wnt11 knockdown. The data represent the mean ± SD of at least three independent experiments; * *p* < 0.05 compared with the control.

**Figure 5:**
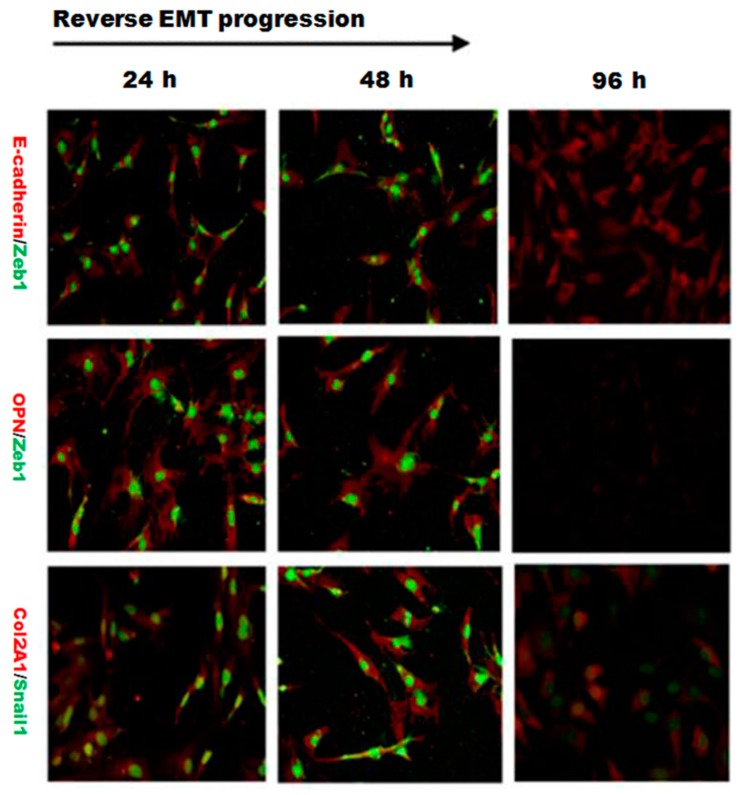
Double immunofluorescence staining showed the dynamic changes in E-cadherin/Zeb1, OPN/Zeb1 and Col2A1/Snail1 after silencing Wnt11. The fluorescence intensity of Zeb1 (green) in the upper two lines showed a dramatic decreasing trend, indicating that the EMT process was significantly inhibited by Wnt11 depletion in PRECs. The fluorescence intensity of OPN (red), Col2A1 (red) and Snail1 (green) also showed a similar attenuating trend in a time-dependent manner (original magnification: ×400).

Wnt signaling pathways are closely related to TGF-β1 and EMT during such diseases, and the non-canonical Wnt11 signaling is directly regulated by TGF-β1, which was a great potential stimulus of EMT. We examined the effect of Wnt11 silencing on the expression of EMT-related and osteochondral factors in PRECs and found that Wnt11 knockdown significantly enhanced the mRNA levels of E-cadherin (Figure 3A) (*p* < 0.05) and remarkably attenuated mRNA levels of mesenchymal markers (Zeb1, Snail1) (Figure 3B,C) (*p* < 0.05) and osteogenic/chondrogenetic markers (Col2A1, Sox9, Runx2 and OPN) (Figure 4A,C,E,G) (*p* < 0.05) in PRECs. Compared with control and Lent-negative groups, the mRNA expressions of Zeb1, Snail1, Col2A1, OPN, Sox9 and Runx2 in Lent-shWnt11 groups were practically decreased from the time point of 24 h, and all were markedly decreased at 48 and 96 h (*p* < 0.05). The expression of mesenchymal and osteogenic/chondrogenetic markers showed the lowest level at 48 and 96 h. Wnt11 knockdown could reverse the reduction of the epithelial marker E-cadherin in PRECs, showing an increased level of E-cadherin mRNA from 24 to 96 h, but without a dramatic change between the two time points. The results from Western blot showed a similar trend in protein expression levels as in mRNA expression levels after silencing the Wnt11 gene in PRECs (Figure 3D,E and Figure 4B,D,F,H). Wnt11 knockdown also reversed the down-expression of E-cadherin at the protein level (Figure 3D). Double immunofluorescence staining (Figure 5) showed that E-cadherin was gradually increased from the 24-h time point and reached the maximum at 96 h. By contrast, Zeb1 in the upper two lines showed a dramatic decreasing trend, indicating that the EMT process was greatly inhibited by Wnt11 depletion in PRECs. In addition, OPN (red), Col2A1 (red) and Snail1 (green) from the lower two lines also showed a similar attenuating trend as Zed1 at the time point of 24 h, and the minimum intensity was observed at 96 h.

### 2.4. EMT Process Was Abrogated by L-Type Calcium Channel Blocker Nifedipine in PRECs

**Figure 6:**
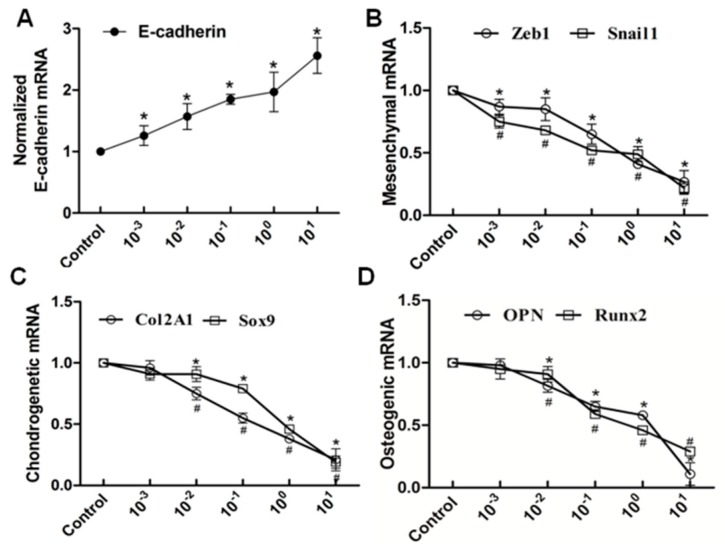
RT-PCR analysis showed that the elevated level of nifedipine attenuated EMT and potential osteochondral differentiation in PRECs. Cells were treated with the indicated concentration of nifedipine for 24 h; RT-PCR was used to examine the expression diversities of epithelial, mesenchymal and osteochondral markers. Data showed the increased expression of E-cadherin (**A**); and decreased expression of Zeb1 and Snail1 (**B**); Col2A1 and Sox9 (**C**) and OPN and Runx2 (**D**) with increasing amounts of nifedipine. * *p* < 0.05 *vs.* the control (without nifedipine) for E-cadherin, Zeb1, Sox9 and Runx2. **^#^** *p* < 0.05 *vs*. the control (without nifedipine) for Snail1, Col2A1 and OPN.

**Figure 7:**
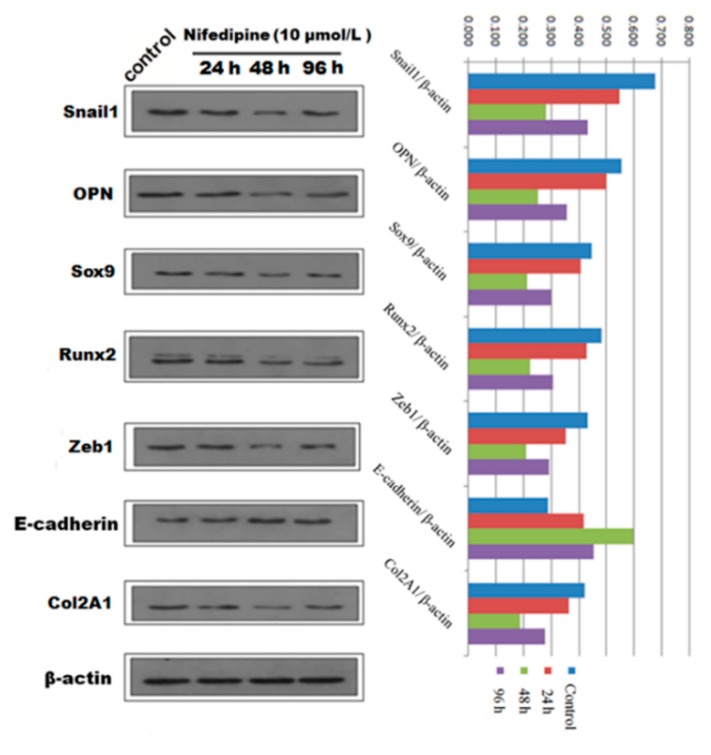
Qualitative and quantitative analysis of Western blot showed the protein expression of EMT and osteochondral-associated markers changed in a time dependent-manner with stimulation by 10 μmol/L nifedipine in PRECs.

We next speculated that high extracellular calcium has a critical effect on EMT and (or) osteogenic/chondrogenetic differentiation. We hypothesized that the L-type calcium channel might be closely associated with calcium influx. We further studied whether blockage of the L-type calcium channel by nifedipine could reverse EMT progression and (or) osteogenic/chondrogenetic differentiation. As shown in Figure 6A, mRNA levels of E-cadherin showed a gradually increasing trend, in line with the increasing of nifedipine, achieving the maximum stimulus effect at 10 μmol/L. In contrast, another six factors in Figure 6B–D showed a gradually decreased trend after incubation of nifedipine in a dose-dependent manner. Further, we confirmed the various phenotypic changes after nifedipine treatment (10 μmol/L) in a time-dependent manner (Figure 7). Progressive upregulation protein levels in E-cadherin were observed from 24–96 h (*p* < 0.05), and Zeb1, Snail1, Col2A1, OPN, Sox9 and Runx2 were decreased at 48 and 96 h after nifedipine treatment.

### 2.5. The EMT Process Was Inhibited by SiCav1.2 in PRECs

It is well known that the L-type calcium channel (LTCC) is a voltage-dependent Ca^2+^ channel, which is composed of a pore forming a α1 subunit, (Cav1.1, Cav1.2, Cav1.3 or Cav1.4) and several associated auxiliary subunits (α2-δ, β, γ). In this part, we examined the effects of Cav1.2 (subunit of L-type calcium channel) silencing on the expression of EMT-related and osteochondral factors in PRECs. Knockdown of the Cav1.2 subunit greatly upregulated the expression of E-cadherin in PRECs, and the relative expression of E-cadherin mRNA increased in a time-dependent manner (Figure S1A). Additionally, we also observed that silencing LTCC by SiCav1.2 significantly reversed the EMT process and osteochondral differentiation (Figure S1B–D). Relative mRNA expression of the mesenchymal marker (Zeb1), chondrogenetic marker (Col2A1) and osteogenic marker (Runx2) also decreased in a time-dependent manner and reached the statistical minimum at 48 and 96 h.

### 2.6. Effects of Incubation of Ca^2+^ and/or TGF-β1 in NRK Cells

**Figure 8:**
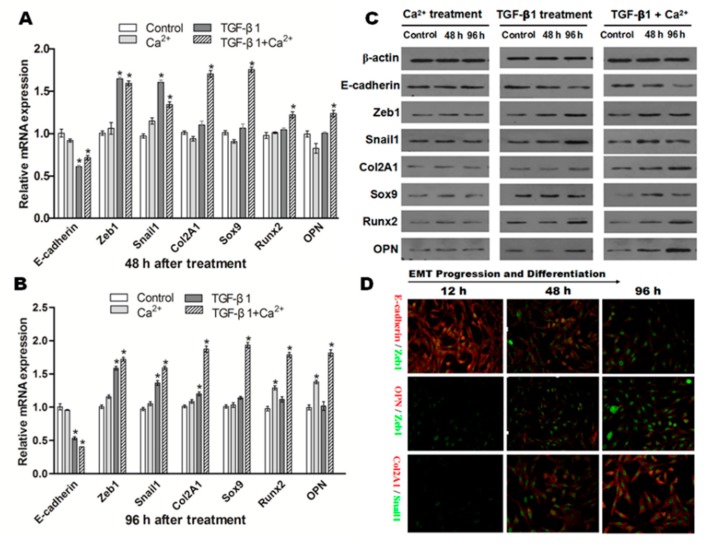
NRK cells induced by TGF-β1, Ca^2+^ or both lead to different differentiation results. Representative data showed the changes on the mRNA expression of all factors related to EMT and osteochondral differentiation in NRK cells exposed to TGF-β1 (10 ng/mL) or/and Ca^2+^ (2.5 mmol/L) after 48 (**A**) and 96 h (**B**); data from three independent experiments (each *n* = 3) were analyzed; significant difference (* *p* < 0.05) relative to the control; (**C**) Western blot analysis confirmed a similar protein expression change as mRNA expression after TGF-β1 or/and Ca^2+^ stimulation; (**D**) Double immunofluorescence staining showed the dynamic changes in E-cadherin/Zeb1, OPN/Zeb1 and Col2A1/Snail1 after co-incubation of TGF-β1 and Ca^2+^ (original magnification: ×200).

The influence of extracellular Ca^2+^ (2.5 mmol) or TGF-β1 (10 ng/mL) on the expression of E-cadherin, Zeb1, Snail1, Col2A1, OPN, Sox9 and Runx2 in NRK cells was detected. As shown in Figure 8A, no significant changes were observed on the mRNA of these factors when NRK cells were exposed to Ca^2+^ (2.5 mmol/L) alone after 48 h. Interestingly, mRNA of Runx2 and OPN were dramatically upregulated in NRK cells exposed to Ca^2+^ (2.5 mmol/L) alone after 96 h (Figure 8B). The consistent trends were observed in terms of protein expression (Figure 8C).

Next, we examined the role of TGF-β1 (10 ng/mL) in EMT and (or) in osteogenic/chondrogenetic differentiation. As shown in Figure 8A,B, the mRNA expression of E-cadherin was significantly downregulated (*p* < 0.05), but mesenchymal markers (Zeb1, Snail1) were significantly increased (*p* < 0.05) in NRK cells after incubation of TGF-β1. Western blot analysis confirmed a similar protein expression change as mRNA expression after TGF-β1stimulation in NRK cells (Figure 8C).

Additionally, the influence of the co-incubation of Ca^2+^ (2.5 mmol/L) and TGF-β1 (10 ng/mL) on the variations of epithelial, mesenchymal and osteochondral markers was also examined. As shown in Figure 8A,B, the results showed that E-cadherin was significantly downregulated in NRK cells after co-incubation of TGF-β1 and Ca^2+^ (*p* < 0.05). In contrast, the mRNA levels of osteochondral markers, including Col2A1, OPN, Sox9 and Runx2, in NRK cells were all upregulated significantly after co-incubation of TGF-β1 and Ca^2+^ (*p* < 0.05). Co-incubation greatly promoted TGF-β1-induced EMT and markedly increased osteochondral differentiation at the protein level (Figure 8C). Furthermore, double immunofluorescence staining (Figure 8D) also showed the dynamic process of E-cadherin/Zeb1, OPN/Zeb1 and Col2A1/Snail1. E-cadherin was gradually downregulated with co-stimulation of TGF-β1 and Ca^2+^ from 12–96 h. However, Zeb1 (green) in the upper two lines gradually increased in a time-dependent manner, suggesting that the EMT process was enhanced in NRK. In addition, OPN (red), Col2A1 (red) and Snail1 (green) from the lower two lines showed an increased trend from 12–96 h. All of these results indicated that co-incubation of TGF-β1 and Ca^2+^ greatly promoted the EMT process and osteochondral differentiation in NRK.

## 3. Discussion

The EMT results from the stimulation of transcriptional factors that alter gene expression and promote the loss of cellular adhesion, triggering a shift in cytoskeletal dynamics and a variation from epithelial morphology and physiology to the mesenchymal phenotype, are shown. Hallmarks of EMT include the loss of expression and function of E-cadherin, reduced tight junction protein and cytokeratin levels and increased expression of mesenchymal markers, such as Zeb1, Snail1, Vimentin and fibronectin. EMT acting as a critical cellular mechanism governs many developmental processes, including embryonic growth, neural crest development, somite dissociation and palate and lip fusion [17]. In addition, EMT also participates in tissue repair, tumor progression and metastasis. Much previous evidence has indicated that EMT is widely involved in the progression of renal tubular interstitial fibrosis (TIF), which subsequently leads to chronic kidney disease (CKD) and, finally, end-stage renal disease (ESRD) [17,18]. Although previous studies have demonstrated the association between nephrolithiasis (kidney stone disease) and renal fibrosis [19,20], the underlying mechanisms in fibrosis-related or EMT progression-associated events in nephrolithiasis formation remain largely unknown. Here, we provided evidence that the nephrolithiasis pathogenesis that initiated in renal tubular epithelial cells was associated with a high calcium ion environment and TGF-β1 stimulation-induced EMT. Consistent with our data, the increased expression levels of TGF-β1 in serum and urine and high levels of intracellular calcium in PRECS were also observed. Thus, we postulated that high calcium served as a critical factor and further could promote re-differentiation of multiple differentiation potential mesenchymal cells into osteochondral-like cells. Decreased epithelial cells markers (E-cadherin) and increased expression of the mesenchymal (Zeb1, Snail1) and osteochondral (Col2A1, OPN, Sox9 and Runx2) phenotypes in PRECs of GHS rats indicated that EMT may already exist in our GHS model and PRECs. Moreover, EMT and related differentiation in PRECs were obviously reversed in the Wnt11 knockdown and nifedipine pretreatment group. Correspondingly, increased levels of Zeb1, Snail1, Col2A1, OPN, Sox9 and Runx2 were observed when co-incubation of TGF-β1 and Ca^2+^ was associated with renal EMT and osteochondral differentiation. These data provided novel insight that the development of nephrolithiasis may result from the stimulation of the abnormal elevated TGF-β1 and Ca^2+^ via the non-canonical Wnt11 signaling pathway and L-type calcium channel, respectively.

TGF-β1 is a well-characterized factor and can induce EMT through intracellular kinase cascades to activate EMT-associated gene, although EMT-targeted genes and their biological effects in renal epithelial cells remain poorly confirmed. These genes activated by TGF-β1 were most associated with the Wnt signaling pathway. Recent studies suggested that Wnt signaling, particularly the canonical Wnt/β-catenin pathway, was involved in EMT- and TGF-β1-mediated fibrosis [21,22]. Wnt11 has diverse functions in regulating cell properties, such as proliferation, migration and differentiation, However, its precise role in different cell types was context-dependent and sometimes contradictory [23,24]. The role of the TGF-β1 signaling cascade was confirmed in the progression of nephrolithiasis by the TGF-β1-dependent RhoA/ubiquitin-proteasome pathway [25] and other renal diseases, such as (UUO)-induced renal fibrosis [26]. However, only a few consolidated views on the relationship between TGF-β1-related Wnt11 and renal stone pathogenesis were reported. Generally, Wnt11 belongs to the Wnt5a subclass, in which it exerts various effects through activation of the non-canonical Wnt signaling pathway. Many data are consistent with Wnt11 activating the non-canonical Wnt signaling pathways through the c-Jun N-terminal kinase (JNK) or CaMKII kinases [27]. Activation of JNK is critical for mediating the Wnt11/TGF-β response, because inhibition of JNK signaling could decrease upregulation of the mesenchymal genes induced by TGF-β and Wnt11. Consistent with our study, using the Wnt11 knockout method, we found that Wnt11-defected PRECs displayed increased expression of E-cadherin and decreased mesenchymal and osteochondral markers. These findings demonstrated that Wnt11 may participate in stone formation related to EMT through the JNK signaling pathway.

Ca^2+^ greatly promoted NRK cell differentiation in the setting of high concentrations of TGF-β1. We speculated that Ca^2+^ may trigger TGF-β1-induced mesenchymal-like cell differentiation. In accordance with this view, our data showed that co-incubation of TGF-β1 and Ca^2+^ greatly promoted the EMT process and osteochondral differentiation in NRK. It has been wide accepted that calcium ions play a critical role as a second messenger in translating extracellular stimulation into intracellular effects [28]. Several research teams have identified voltage-dependent calcium channels, involving the L-type calcium channel (CaV1.2), in renal epithelial cells [29,30,31]. The L-type Ca^2+^ channels are expressed ubiquitously in the kidney, including proximal tubules, medullary collecting ducts, cortical thick ascending limbs and distal convoluted tubules, representing an important role of Ca^2+^. Our data further confirmed that blockage of the L-type calcium channel by nifedipine and SiCav1.2 (refer to Figure S1) could lead to increased E-cadherin, but decreased mesenchymal and osteochondral gene expression, which indicated that the L-type calcium channel acts as an important intermediary in the development of kidney stone formation associated with calcium influx. Additionally, we considered that calcium influx through channels is more critical than calcium released from intracellular ER; It is well known that Ca^2+^ entering cells via store-operated channels (SOCs) affects most cell functions. Thus, we also detected the functions of the IP3-mediated store-operated calcium (STIM1/ORAI1) channel, and we found a similar phenomenon that the blockage of STIM1 and ORAI1 by inhibitors (2-APB and SKF96365) or RNAi also attenuated the EMT process and osteochondral differentiation significantly (refer to Figure S2). Moreover, we also detected the co-effect of nifedipine and Wnt11 depletion in PRECs, and results were shown in Figure S3.

Taken together, we demonstrated that TGF-β1 and Ca^2+^ are upregulated in GHS rats and PRECs, respectively. Wnt11 depletion and the L-type Ca^2+^ channel blocker greatly reversed EMT progression and osteochondral differentiation in PRECs. Co-incubation of extracellular Ca^2+^ and TGF-β1 promotes EMT and osteochondral differentiation in NRK cells. This study suggests that TGF-β1 and Ca^2+^ have synergic effects to promote EMT and osteochondral differentiation via Wnt11 and the L-type calcium channel, respectively. It supplied a novel view that the pathogenesis of calcium stone development is associated with bone formation.

## 4. Materials and Methods

### 4.1. Animals and Cells Preparation

The animal model of genetic hypercalciuric stone-forming (GHS) rats has been established for 27 generations in our panel, with a body weight of 200–260 g. Steady hereditary characteristics of hypercalciuric rats were screened from 7 generations. Sprague–Dawley (SD) rats with urine calcium excretion >1.5 mg/24 h on twice successive 24 h urine collections were defined as GHS rats [14]. All animal procedures were approved by the Animal Care Committees of Huazhong University of Science and Technology. A total of 15 common SD rats matched with GHS rats on body weight and age served as the normal control (NC). Blood samples were collected as previously [32,33], and sterilized 24-h urine samples were also collected for further detection until the time the rats were euthanized. Primary renal tubular epithelial cells (PRECs) were obtained from the renal specimens of GHS rats (8 weeks), as our previous report [14]. The normal rat (Rattus norvegicus) kidney epithelial cell line NRK (Cell Bank, Shanghai, China) was cultured in DMEM, supplemented with 10% fetal bovine serum at 37 °C in a humidified atmosphere of 5% CO_2_. Serum-free medium was used when serum starvation was necessary.

### 4.2. Enzyme-Linked Immunosorbent Assay

For the determination of serum or urine TGF-β1 concentrations, the ELISA (R & D systems Inc., Minneapolis, MN, USA) was performed according to the manufacturer’s instructions.

### 4.3. Evaluation of Intracellular Ca^2+^ by Flow Cytometry Assay

The PRECs and NRK cells (containing more than 1 × 10^6^ cells) without any stimulation were trypsinized, centrifuged and fixed with prechilled 75% ethanol at 4 °C for more than 18 h. Cells were loaded with 1 μmol/L Fluo-3 AM (Fluo-3 AM calcium assay kit, Invitrogen, Carlsbad, CA, USA) at 37 °C for 30 min, according to the manufacturer’s instructions. Cells were suspended in HBSS (Hank’s Balanced Salt Solution) after being washed three times. At least five randomly-chosen cells were analyzed by a flow cytometry device (BD Bioscience, San Jose, CA, USA) at 488 nm. All data were analyzed using Windows Multiple Document Interface for Flow Cytometry (WinMDI, Version 2.9, Joseph Trotter©, The Scripps Research Institute, La Jolla, CA, USA).

### 4.4. Real-Time RT PCR

Total RNA extraction was performed using TRIzol^®^ Reagent (Invitrogen, Carlsbad, CA, USA). The cDNA synthesis (2 μg of total RNA) was performed by using Omnisoript Reverse Transcriptase for the first-strand cDNA synthesis (Qiagen, Hilden, Germany) and the oligo-dT primers according to the manufacturer’s protocol. The mRNA levels of E-cadherin, CK19, Zeb1, Snail1, Col2A1, Sox9, Runx2, OPN and housekeeping gene β-actin were analyzed by quantitative PCR on a Rotor Gene 3000 (Corbett Research, New South Wales, Australia) using SYBR Premix Ex Taq II (Takara, Japan). The amplification reactions were performed under the following conditions: 95 °C denaturing for 10 min, following by 40 cycles of 95 °C for 15 s, 59 °C for 15 s and 72 °C for 30 s. The primer sequences are described as in Table 1. The outcomes of *C*_t_ values were calculated by the comparative *C*_t_ (ΔΔ*C*_t_) method. The fold difference was calculated as 2^−ΔΔ*C*t^.

**Table 1:** Sequences of rat primers for real-time RT PCR.

Gene	Forward Primer (5′–3′)	Reverse Primer (5′–3′)
*E-cadherin*	GAGGCCAAGCAGCAGTACATT	CGATACGTGATCTTCTGTTCCA
*CK19*	TAATGGCGAGCTGGAGGTGA	TCGATCTGTAGGACTATCTTGGAG
*Zeb1*	CCGCAGGGTTACTCTTGTGT	TTGGCACTTGGTGGGACTAC
*Snail1*	CGGAAGCCCAACTATAGCGA	AGAGTCCCAGATGAGGGTGG
*Col2A1*	GAAGAGCGGAGACTACTGGATTG	CTGGACGTTAGCGGTGTTGGGAG
*Sox9*	CACCAGAACTCCGGCTCCTA	TGTGGGTCTGCGGGATGGAA
*Runx2*	AGCCACCTTCACTTACACCC	CCATTGGGAACTGATAGGAC
*OPN*	GGTTTGCTTTTGCCTGTTCG	GTGGCTACAGCATCTGAGTGTTTG
*OCN*	GTGCAGACCTAGCAGACACCAT	TTCACCACCTTACTGCCCTC
*β-actin*	CACGATGGAGGGGCCGGACTCATC	TAAAGACCTCTATGCCAACACAGT

### 4.5. Western Blot Analysis

Extracts of total cellular proteins were prepared by scraping the cells into Mammalian Protein Extraction Reagent (Thermo Scientific Pierce, No. 78503, Shanghai, China). The protein samples were separated on the 12% SDS-PAGE and electrotransferred onto polyvinylidene difluoride-nitrocellulose membranes (Immobilon-P; Millipore Corporation, Bedford, MA, USA). The following primary antibodies were used: E-cadherin (SC-8426, Santa Cruz Biotechnology, Inc., Santa Cruz, CA, USA), Cytokeratin19 (CK19) (SC-56371, Santa Cruz Biotechnology, Inc.), Zeb1 (ABIN755113, Antibodies-online, Shanghai, China), Snail1 (ABIN182776, Antibodies-online, Shanghai, China), Sox9 (SC-20095, Santa Cruz Biotechnology, Inc.), Col2A1 (SC-52658, Santa Cruz Biotechnology, Inc.), Runx2 (No. 12556, Cell Signaling Technology, Boston, MA, USA), OPN (SC-21742, Santa Cruz Biotechnology, Inc.), STIM1 (SC-166541, Santa Cruz Biotechnology, Inc.), ORAI1 (SC-68895, Santa Cruz Biotechnology, Inc.). HRP-conjugated goat anti-mouse or goat anti-rabbit secondary antibodies (Boster, BA1051, Wuhan, China) were used and detected by electrochemiluminescence (Amersham Pharmacia Biotech, Aylesbury, UK).

### 4.6. Immunofluorescence Staining

Cells were cultured on glass coverslips, fixed in 4% paraformaldehyde (PFA) for 20 min at room temperature, permeabilized with 0.5% Triton X-100 for 20 min and then incubated with corresponding antibodies at 4 °C overnight. For the immunofluorescence double staining experiments, appropriate secondary antibodies (diluted 1:50) were used. Sections were mounted with vectashield hard set medium (Vector Laboratories, Shanghai, China) and visualized under a fluorescence microscope (Olympus BX51, Olympus Corporation, Tokyo, Japan).

### 4.7. shRNA-Mediated Gene Knockdown

PRECs were seeded on the 6-well plate 24 h before transfection. The lentiviral vector containing small hairpin RNA (shRNA) targeting Wnt11 was constructed by Genechem Co., Ltd. (Shanghai, China). The targeting sequence of shRNA was 5′-CGTCTACACAACAGTGAAG-3′. Lentivirus was added to 8 μg/mL Polybrene and kept overnight. Puromycin was added for selection for 10 days before experiments. Wnt11 shRNA lentivirus was used to knock down in PRECs. Next, a GFP tag was encoded in the vector sequence, and GFP-lentivirus served as a negative control for Wnt11 RNAi-lentivirus. The sequence of the vector with the shRNA inserted was identified using sequencing as designed. The immune assay was used to titer the virus. The final lentiviral vector titers were determined to contain 2 × 10^8^ Tu/mL.

### 4.8. Treatment of Nifedipine in PRECs and Incubation of Ca^2+^ (Calcium Chloride) and/or TGF-β1 in NRK Cells

PRECs were cultured to the third generation before treatment with nifedipine (Sigma Corporation, USA) at different concentrations, including 10^−3^, 10^−2^, 10^−1^, 1 and 10 μmol/L for 96 h, respectively. NRK cells were at the third generation prior to treating with calcium chloride (2.5 mmol/L) (Fortuna Chemical Co., Wuhan, China) and/or TGF-β1 (10 ng/mL) (PeproTech Inc., Rocky Hill, NJ, USA). Cycloheximide (5 μg/mL, Sigma-Aldrich, St. Louis, MO, USA) was added to inhibit translation.

### 4.9. Orai1 Gene and Cav1.2 Subunit of L-Type Ca^2+^ Channel Knockdown by siRNA

PRECs were grown in DMEM and 10% fetal bovine serum supplemented with 100 U/mL penicillin G and 100 μg/mL streptomycin. Cells were allowed to grow to ~70% confluence and transfected with the required DNA at a concentration of 1 μg/mL, using Lipofectamine 2000 and protocols supplied by the manufacturer (Invitrogen). Knockdown experiments were carried out by transfection of Orai1 siRNA (targeting sequence: 5′-GCCAUAAGACUGACCGACA-3′) or control siRNA (Dharmacon, Lafayette, CO, USA), transfected at a concentration of 0.8 nmol/mL, using DharmaFECT Duo reagent and protocols supplied by the manufacturer. The Cav1.2 subunit of the L-type Ca^2+^ channel was knocked down by siRNA designed to target the Cav1.2 subunit (SiCav1.2, Dharmacon) using the nucleofection protocol described by Amaxa. The control RNA was a scrambled sequence and was provided by Ambion. Real-time RT PCR were utilized to detect the mRNA levels of E-cadherin, Zeb1, Col2A1 and Runx2 in PRECs when cells were treated by SiCav1.2 for 48 h.

### 4.10. Statistical Analysis

Comparisons among multiple groups were performed using analysis of variance (ANOVA) followed by Student’s *t*-tests using SPSS 13.0 (SPSS, Chicago, IL, USA). Student’s *t*-test was used to compare two independent samples. Values are expressed as the mean ± SD. * *p* < 0.05 was considered to indicate a statistically-significant difference.

## 5. Conclusions

Our study suggested that TGF-β1 and Ca^2+^ have synergic effects to promote EMT and osteochondral differentiation via Wnt11 and the L-type calcium channel in kidney stone model and corresponding primary renal epithelial cells. It supplied a novel view that the pathogenesis of calcium stone development is associated with bone formation.

## Supplementary Materials

Supplementary materials can be found at http://www.mdpi.com/1422-0067/16/07/16313/s1.

## References

[1] Worcester E.M., Coe F.L. (2009). Does idiopathic hypercalciuria trigger calcium-sensing receptor-mediated protection from urinary supersaturation?. J. Am. Soc. Nephrol..

[2] Yao J.J., Bai S., Karnauskas A.J., Bushinsky D.A., Favus M.J. (2005). Regulation of renal calcium receptor gene expression by 1,25-dihydroxyvitamin D3 in genetic hypercalciuric stone-forming rats. J. Am. Soc. Nephrol..

[3] Walker V., Cook P., Griffin D.G. (2014). Male hypercalciuric stone formers with low renal calcium reabsorption. J. Clin. Pathol..

[4] Worcester E.M., Bergsland K.J., Gillen D.L., Coe F.L. (2013). Evidence for increased renal tubule and parathyroid gland sensitivity to serum calcium in human idiopathic hypercalciuria. Am. J. Physiol. Ren. Physiol..

[5] Yoon V., Adams-Huet B., Sakhaee K., Maalouf N.M. (2013). Hyperinsulinemia and urinary calcium excretion in calcium stone formers with idiopathic hypercalciuria. J. Clin. Endocrinol. Metab..

[6] Berridge M.J., Lipp P., Bootman M.D. (2000). The versatility and universality of calcium signalling. Nat. Rev. Mol. Cell Biol..

[7] Boyette L.B., Creasey O.A., Guzik L., Lozito T., Tuan R.S. (2014). Human bone marrow-derived mesenchymal stem cells display enhanced clonogenicity but impaired differentiation with hypoxic preconditioning. Stem Cells Transl. Med..

[8] Ho I.A., Toh H.C., Ng W.H., Teo Y.L., Guo C.M., Hui K.M., Lam P.Y. (2013). Human bone marrow-derived mesenchymal stem cells suppress human glioma growth through inhibition of angiogenesis. Stem Cells.

[9] Zhang L., Yang C., Li J., Zhu Y., Zhang X. (2014). High extracellular magnesium inhibits mineralized matrix deposition and modulates intracellular calcium signaling in human bone marrow-derived mesenchymal stem cells. Biochem. Biophys. Res. Commun..

[10] Sun S., Liu Y., Lipsky S., Cho M. (2007). Physical manipulation of calcium oscillations facilitates osteodifferentiation of human mesenchymal stem cells. FASEB J..

[11] Zeisberg E.M., Tarnavski O., Zeisberg M., Dorfman A.L., McMullen J.R., Gustafsson E., Chandraker A., Yuan X., Pu W.T., Roberts A.B. (2007). Endothelial-to-mesenchymal transition contributes to cardiac fibrosis. Nat. Med..

[12] Yi P., Gao S., Gu Z., Huang T., Wang Z. (2014). P44/WDR77 restricts the sensitivity of proliferating cells to TGF β signaling. Biochem. Biophys. Res. Commun..

[13] Zhang P., Cai Y., Soofi A., Dressler G.R. (2012). Activation of Wnt11 by transforming growth factor-β drives mesenchymal gene expression through non-canonical Wnt protein signaling in renal epithelial cells. J. Biol. Chem..

[14] Jia Z., Wang S., Tang J., He D., Cui L., Liu Z., Guo B., Huang L., Lu Y., Hu H. (2014). Does crystal deposition in genetic hypercalciuric rat kidney tissue share similarities with bone formation?. Urology.

[15] He D., Wang S., Jia Z., Cui L., Lu Y., Hu H., Qin B. (2015). Calcium ions promote primary renal epithelial cell differentiation into cells with bone-associated phenotypes via transforming growth factor-β1-induced epithelial-mesenchymal transition in idiopathic hypercalciuria patients. Mol. Med. Rep..

[16] Moe O.W., Bonny O. (2005). Genetic hypercalciuria. J. Am. Soc. Nephrol..

[17] Strutz F.M. (2009). EMT and proteinuria as progression factors. Kidney Int..

[18] Liu Y. (2004). Epithelial to mesenchymal transition in renal fibrogenesis: Pathologic significance, molecular mechanism, and therapeutic intervention. J. Am. Soc. Nephrol..

[19] Iseki K. (2008). Chronic kidney disease in Japan. Int. Med..

[20] Saucier N.A., Sinha M.K., Liang K.V., Krambeck A.E., Weaver A.L., Bergstralh E.J., Li X., Rule A.D., Lieske J.C. (2010). Risk factors for CKD in persons with kidney stones: A case-control study in Olmsted County, Minnesota. Am. J. Kidney Dis..

[21] Zacharias A.L., Gage P.J. (2010). Canonical Wnt/β-catenin signaling is required for maintenance but not activation of Pitx2 expression in neural crest during eye development. Dev. Dyn..

[22] He W., Dai C., Li Y., Zeng G., Monga S.P., Liu Y. (2009). Wnt/β-catenin signaling promotes renal interstitial fibrosis. J. Am. Soc. Nephrol..

[23] Dwyer M.A., Joseph J.D., Wade H.E., Eaton M.L., Kunder R.S., Kazmin D., Chang C.Y., McDonnell D.P. (2010). WNT11 expression is induced by estrogen-related receptor α and β-catenin and acts in an autocrine manner to increase cancer cell migration. Cancer Res..

[24] Cha S.W., Tadjuidje E., White J., Wells J., Mayhew C., Wylie C., Heasman J. (2009). Wnt11/5a complex formation caused by tyrosine sulfation increases canonical signaling activity. Curr. Biol..

[25] Kanlaya R., Sintiprungrat K., Thongboonkerd V. (2013). Secreted products of macrophages exposed to calcium oxalate crystals induce epithelial mesenchymal transition of renal tubular cells via RhoA-dependent TGF-β1 pathway. Cell. Biochem. Biophys..

[26] Vuruskan H., Caliskan Z., Kordan Y., Ozakin C., Yavascaoglu I., Oktay B. (2005). Elevated plasma concentrations of transforming growth factor-β 1 in patients with unilateral ureteral obstruction. Urol. Res..

[27] Westfall T.A., Brimeyer R., Twedt J., Gladon J., Olberding A., Furutani-Seiki M., Slusarski D.C. (2003). Wnt-5/pipetail functions in vertebrate axis formation as a negative regulator of Wnt/β-catenin activity. J. Cell Biol..

[28] Jones T.J., Nauli S.M. (2012). Mechanosensory calcium signaling. Adv. Exp. Med. Biol..

[29] Monteil A., Chemin J., Bourinet E., Mennessier G., Lory P., Nargeot J. (2000). Molecular and functional properties of the human α_1G_ subunit that forms T-type calcium channels. J. Biol. Chem..

[30] Andreasen D., Jensen B.L., Hansen P.B., Kwon T.H., Nielsen S., Skott O. (2000). The α_1G_-subunit of a voltage-dependent Ca^2+^ channel is localized in rat distal nephron and collecting duct. Am. J. Physiol. Ren. Physiol..

[31] Zhao P.L., Wang X.T., Zhang X.M., Cebotaru V., Cebotaru L., Guo G., Morales M., Guggino S.E. (2002). Tubular and cellular localization of the cardiac L-type calcium channel in rat kidney. Kidney Int..

[32] Tiwari S., Halagappa V.K., Riazi S., Hu X., Ecelbarger C.A. (2007). Reduced expression of insulin receptors in the kidneys of insulin-resistant rats. J. Am. Soc. Nephrol..

[33] Song J., Hu X., Riazi S., Tiwari S., Wade J.B., Ecelbarger C.A. (2006). Regulation of blood pressure, the epithelial sodium channel (ENaC), and other key renal sodium transporters by chronic insulin infusion in rats. Am. J. Physiol. Ren. Physiol..

