# Supplementary material for: The Wnt11 Signaling Pathway in Potential Cellular EMT and Osteochondral Differentiation Progression in Nephrolithiasis Formation
Source: Int J Mol Sci. 2015 Jul 17;16(7):16313–29. doi: 10.3390/ijms160716313 (PMC4519952; doi:10.3390/ijms160716313)
Supplement: Supplementary file 1 [file ijms-16-16313-s001.pdf]

Supplementary Information

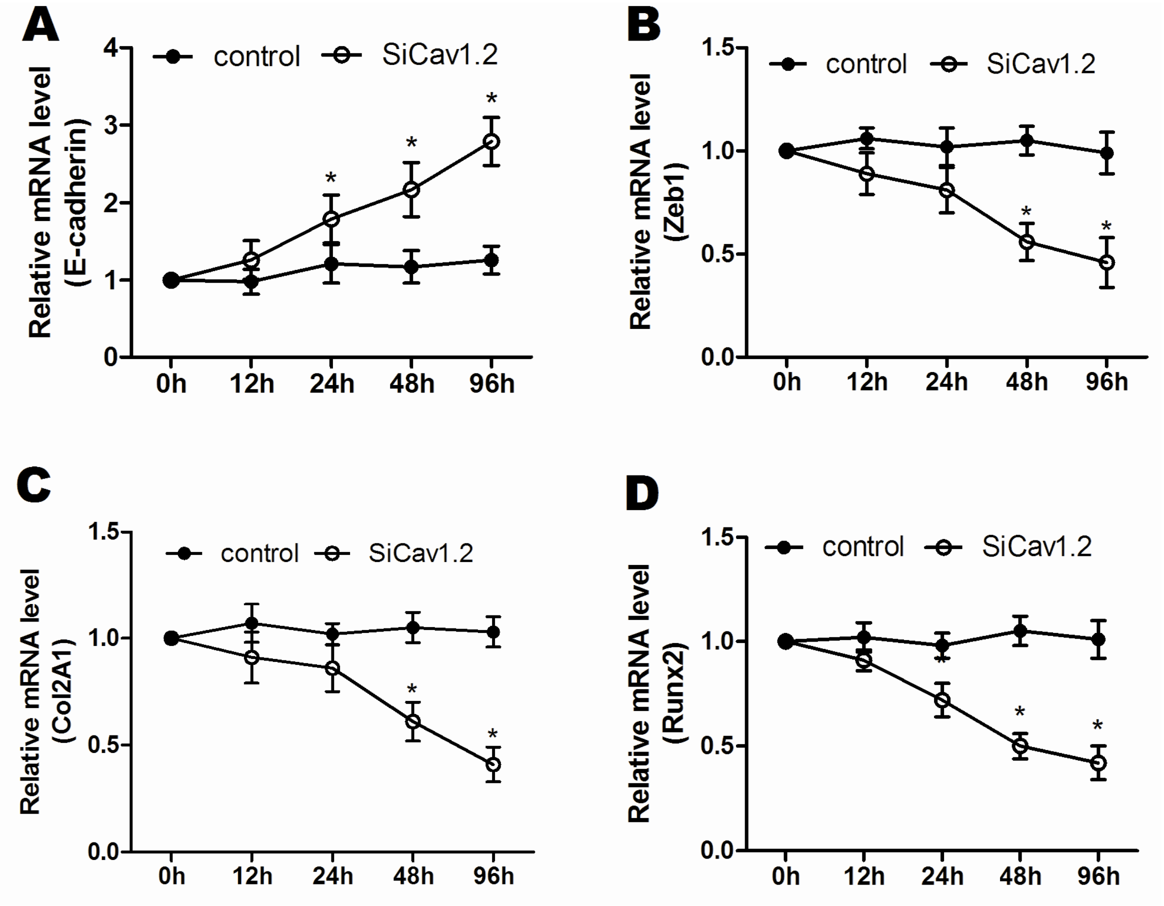

Figure S1. The inhibition of Cav1.2 subunit of L-type calcium channel by RNAi have the potential inhibitory effects for cellular EMT and osteogenic/chondrogenetic differentiation in PRECs. (**A**) The relative expression of E-cadherin mRNA significantly increased in a time dependent-manner after Cav1.2 subunit depletion in PRECs. Additionally, relative expression of Zeb1; (**B**) Col2A1; (**C**) Runx2; (**D**) were also detected, and showed the decreasing trend in a time dependent-manner, and reached to the statistical minimum at 48 and 96 h. All data from three independent experiments (n = 3) were analyzed, significant difference (* $p < 0.05$) relative to control.

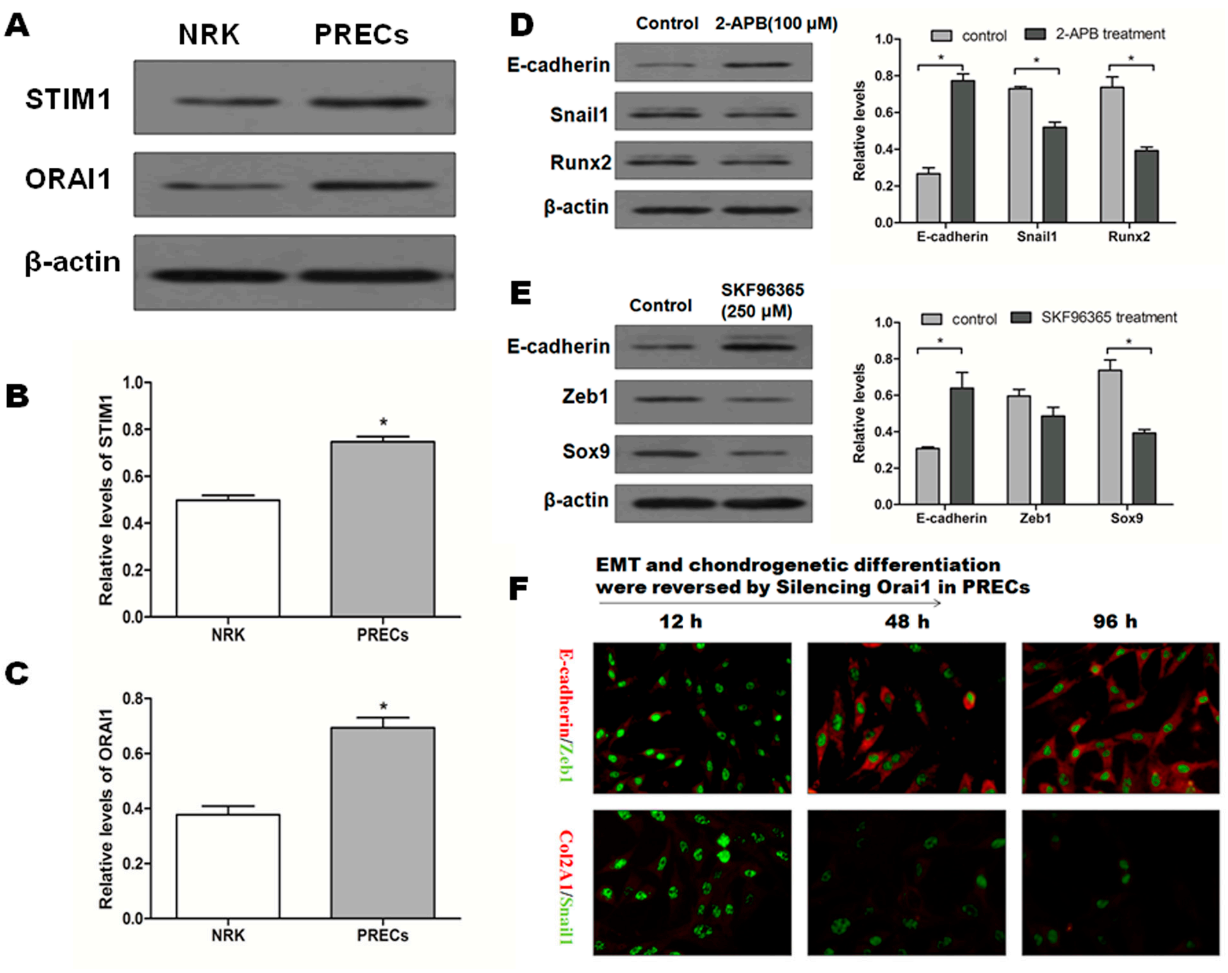

Figure S2. The IP3-mediated store-operated calcium channels (SOCs) have potential roles in EMT process and relevant differentiation in PRECs. The protein expressions of STIM1 and ORAI1 were analyzed by western blot respectively. (**A**–**C**) The levels of STIM1/ORAI1 in PRECs were significantly upregulated comparing with those of NRK cells (control) (n = 3); (**D**,**E**) The expression of E-cadherin in PRECs increased significantly when cells were incubated with 2-APB (100 μmol/L) and SKF96365 (250 μmol/L), the osteogenic/chondrogenetic markers including Runx2 and Sox9 were also inhibited by 2-APB and SKF96365 respectively (n = 3), the level of mensenchymal marker Snail1 in PRECs decreased statistically when cells were treated by 2-APB (n = 3). In addition, Double immunofluorescence staining showed Orai1 gene silencing attenuated the potential EMT and chondrogenetic differentiation in PRECs, and the dynamic changes of E-cadherin/Zeb1 and Col2A1/Snail1 were shown in (**F**) (Original magnification ×400). Significant difference (* $p < 0.05$) relative to control (NRK cells).

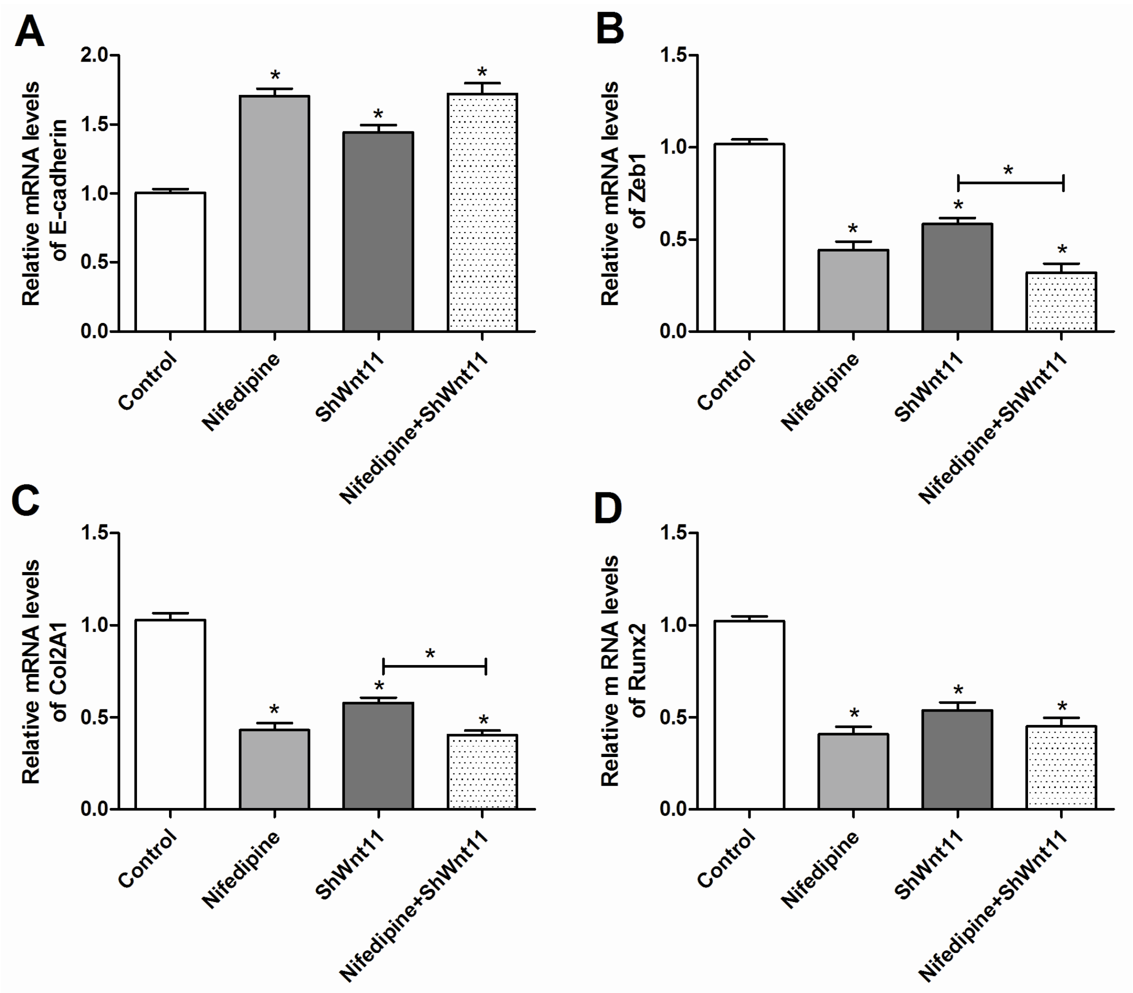

Figure S3. Quantitative analysis of RT-PCR showed the mRNA expression of EMT (**A**,**B**) and osteochondral (**C**,**D**) markers changed significantly with treatment of nifedipine (10 μmol/L) or/and Wnt11 depletion in PRECs. * $p < 0.05$ *vs.* the control.
